# Tuning the Supramolecular Structures of Metal-Free Porphyrin via Surfactant Assisted Self-Assembly to Enhance Photocatalytic Performance

**DOI:** 10.3390/nano9091321

**Published:** 2019-09-15

**Authors:** Jinrong Lu, Zihan Li, Weijia An, Li Liu, Wenquan Cui

**Affiliations:** College of Chemical Engineering, Hebei Key Laboratory for Environment Photocatalytic and Electrocatalytic Materials, North China University of Science and Technology, Tangshan 063210, China; lujinrong@ncst.edu.cn (J.L.); zihan0unicorn@163.com (Z.L.); anweijia@ncst.edu.cn (W.A.); chemll@126.com (L.L.)

**Keywords:** metal-free porphyrin, self-assembly, aggregation mode, photocatalysis

## Abstract

Metal-free porphyrin with good planarity is beneficial to π–π stack interactions, which promotes electron coupling and the separation and transfer of photogenerated carriers. It is necessary to develop metal-free porphyrin-based photocatalysts and exploit the photocatalytic mechanism. Herein, metal–free porphyrin (5,10,15,20-tetrakis(4-carboxyphenyl)porphyrin, TCPP) was self-assembled through an acid-based neutralization reaction and mixing dual-solvents under surfactants to form different aggregates. Morphology structures, optical and optoelectronic properties of the TCPP aggregates were characterized in detail. TCPP self-assemblies showed higher photocatalytic activities for the degradation of phenol under visible light than untreated TCPP powders, and the aggregates of nanorods formed through the acid-based neutralization reaction in the presence of hexadecyl trimethyl ammonium bromide (CTAB) possessed 2.6 times more activity than the nanofiber aggregates formed through mixing dual-solvents. It was proved that self-assembly methods are crucial for controlling the aggregation of porphyrins to form different aggregations, which have a profound impact on the photocatalytic activity.

## 1. Introduction

Photocatalysis is an effective technology for storing and transforming solar energy, which would help to solve environmental pollution caused by water contamination [1]. The key to this technology is to develop photocatalysts with high efficiency, stability, environmental friendliness and abundant sources of raw material. Organic materials as photocatalysts possess many advantages, such as excellent light absorption, tunability in structure, and rich resources [2,3]. Their use can avoid the introduction of metal contamination, therefore, utilization of organic photocatalysts such as graphitic carbon nitride (g-C_3_N_4_) [4] and perylene diimide derivatives (PDIs) [5] has attracted attention to achieve splitting water, the reduction of carbon dioxide and degradation of pollutants under visible light. However, photogenerated carriers are easy to recombine in organic photocatalysts and the photocatalytic efficiency is low [6]. In addition, organic molecules have poor light and thermal stability. So, to solve these problems, the organic molecules with π-conjugated structure are usually arranged by intermolecular non-covalent bonding to form supramolecular nanostructures, which capture photons more effectively and prolong the excited state electron-holes in ordered aggregates [7,8,9]. This has proved to be an effective strategy for achieving efficient photocatalysis using supramolecular structures of organic small molecule semiconductors [10,11].

Porphyrin molecules with their planar aromatic macrocyclic structure are the centers of photosynthetic reactions in plants, have strong visible absorption, and are mainly used as light-trapping antenna molecules and cocatalysts in photocatalytic systems [12]. Based on the rigid molecular skeleton and abundant substituents, aggregates of porphyrins are easily formed using self-assembly techniques [13] such as reprecipitation, surfactant assisted crystallization and solvent phase volatilization. The aggregates not only broaden the light absorption range, but also enhance the stability of the porphyrin photocatalyst due to the geometrical constraints of the rigid frame [14]. Furthermore, the mode of molecules aggregation and morphology of nanostructures are very essential to the separation and transmission of photogenerated charges [15,16]. When the porphyrin is assembled into a crystal structure, higher charge mobility can be obtained, and is an important prerequisite for photocatalytic reactions [17,18,19,20].

In the past, porphyrin-based photocatalysts mainly concentrated on metallo-porphyrins and the mechanism of the molecular and aggregation mode of photocatalysis in porphyrin aggregates is still unclear. On the other hand, the excellent planarity of metal-free porphyrin is beneficial to the π-π stack interactions, which promotes electron coupling and facilitates separation and transfer of photogenerated carriers [18]. Herein, a metal–free porphyrin (5,10,15,20-tetrakis(4-carboxyphenyl) porphyrin, TCPP) was self-assembled through an acid-base neutralization reaction in the presence of hexadecyl trimethyl ammonium bromide (CTAB) as a surfactant and mixing dual-solvents under ethylene glycols (EG) as a surfactant. The aggregates were used as photocatalysts for degradation of phenol. We showed that self-assembly methods are crucial for controlling the aggregation of porphyrins to form different aggregations, which have a profound effect on the photocatalytic properties of the material.

## 2. Materials and Methods

### 2.1. Materials

5,10,15,20-tetrakis(4-carboxyphenyl)porphyrin (97% purity, TCPP) was purchased from Aladdin Reagent (Shanghai, China). Sodium hydroxide, ethylene glycol (EG), anhydrous sodium sulfate and tetrahydrofuran (THF) were provided by Yongda Chemical Reagent Co., Ltd. (Tianjin, China). HCl was purchased from Kaixin Chemical Industry Co., Ltd. Hexadecyl trimethyl ammonium bromide (CTAB) was purchased from Xingfu Fine Chemical Research Institute (Tianjin, China). All the chemical reagents used were of analytical purity and not further purified.

### 2.2. Characterization of the Materials

The X-ray diffraction (XRD) was tested by D8 Advance X-Ray Diffractometer (Buker, Germany). Field emission scanning electron microscopy (SEM) was tested by Hitachi S-4800 (Tokyo, Japan). Transmission electron microscopy (TEM) was tested by Hitachi HT-7700 (Tokyo, Japan). Dynamic light scattering (DLS) was measured by the Malvern Mastersizer 3000 instrument (Malvern, Britain). Optical absorption spectra were measured by UV−vis spectrophotometer (UV-9000S, Shanghai, China). The FT-IR (Fourier Transform Infrared) spectra were obtained by the VERTEX70 spectrometer (Buker, Germany). The CHI660E electrochemical workstation (Shanghai Chenhua Co., Ltd., Shanghai, China) with a 500 W xenon lamp (420 nm filter) and three-electrode quartz cells were used to measure the electrochemical properties of the aggregates. Electron paramagnetic resonance (ESR) spectra was tested on the CTAB-TCPP samples containing 2,2,6,6-Tetramethyl-4-piperidone (TEMP) as a probe of ^1^O_2_ under dark and visible light by the ESR spectrometer (BrukerA300, Buker, Germany) at rt.

### 2.3. Synthesis of Self-Assembled TCPP

#### 2.3.1. The Self-Assembly Method through Acid-Base Neutralization in the Presence of CTAB

TCPP (0.0190 g) was dissolved in 2.5 mL of NaOH (0.2 M) to form the TCPP/NaOH solution (0.01 M). Then, 0.5 mL of TCPP/NaOH solution was rapidly injected into 9.5 mL of aqueous solution containing CTAB (2.5 mM) and HCl (12 mM), which was stirred continuously. The mixed solutions needed to be stirred (1020 rpm) at room temperature (25 °C) for 6 h. Finally, the mixture was freeze dried to obtain the self-assembled TCCP (CTAB-TCPP) as solid catalyst.

#### 2.3.2. The Self-Assembly Method through Acid-Base Neutralization without CTAB

TCPP (0.0190 g) was dissolved in 2.5 mL of NaOH (0.2 M) to form the TCPP/NaOH solution (0.01 M). Then, 0.5 mL of TCPP/NaOH solution was rapidly injected into 9.5 mL of aqueous solution containing HCl (12 mM) under stirring. The mixed solutions were stirred (1020 rpm) at room temperature (25 °C) for 6 h. Finally, the mixture was freeze dried to obtain the self-assembled TCCP (CTAB-TCPP) as solid catalyst.

#### 2.3.3. The Self-Assembly Method through Mixing Dual-Solvents under Ethylene Glycols (EG)

TCPP (0.0032 g) was dissolved in 4.925 mL of THF solution (0.8 mM). Then 73.625 mL of H_2_O mixed with surfactants ethylene glycols (1.25 mL) was slowly added to the stirred TCPP/THF solution. The produced self-assembled TCCP (EG-TCPP) as flocculation was collected by filtration and freeze-drying.

### 2.4. Evaluation of Photocatalytic Activity

Photocatalytic performance of catalysts under visible light was studied using phenol as the model pollutant. The photocatalytic performance was tested by a multi-tube photocatalytic reactor fromXPA-7(Xujiang Machine Factory, Nanjing, China). To keep the reaction solution temperature at a constant of 25 ± 2 °C, the reaction device was connected to the cooling instrument. A 350 W xenon lamp with a cut-off filter of 420 nm and with about 25 mW/cm^2^ light intensity tested by an optical power meter (CEL-NP2000, Beijing, China) was employed as a visible light for irradiation.

First, 30 mL of 5 ppm phenol was added to a 50 mL reaction tube. 10 mg of TCPP aggregates as catalysts was then added to the phenol solution at a temperature of 25 °C under magnetic stirring. Before the photocatalytic degradation experiment, the adsorption performance of every photocatalyst with phenol was tested under light-off conditions. The reaction without light was for 1 h with stirring to eliminate the adsorption effects. Then, the light was turned on for photocatalytic degradation of phenol. One mL of the degradation solution was taken out at every time interval. The obtained solution was filtered by a 0.22 μm membrane and the concentration of phenol was detected by high performance liquid chromatography (HPLC).

Free radical trapping experiments were carried out to trace the active species of photocatalytic degradation of phenol. The trapping agents used in the experiment were isopropanol (IPA) for quenching •OH, p-benzoquinone (p-BQ) for quenching •O_2−_, and ethylene diamine tetraacetic acid disodium salt (EDTA-2Na) for quenching h^+^. The trapping experiment was performed as the same as that of removing phenols without these scavengers. The determination of the phenol removal rate was also by high performance liquid chromatography (HPLC).

### 2.5. Electrochemical Measurement

Photoelectrochemical measurements mainly proved the separation efficiency of photo generated electrons and holes byCHI660B electrochemical workstation. The used counter electrode was Pt wire, the reference electrode was a saturated calomel electrode (SCE), and the catalyst was coated in ITO glass as a working electrode to form the three-electrode system. A 500 W xenon lamp with a 420 nm cutoff filter was used as a light source and the electrolyte solution was Na_2_SO_4_ (0.1 mol/L) solution.

## 3. Results and Discussion

### 3.1. Self-Assembly Properties of TCPP to Form Different Aggregates and the Characterization of Their Structures

**Figure 1:**
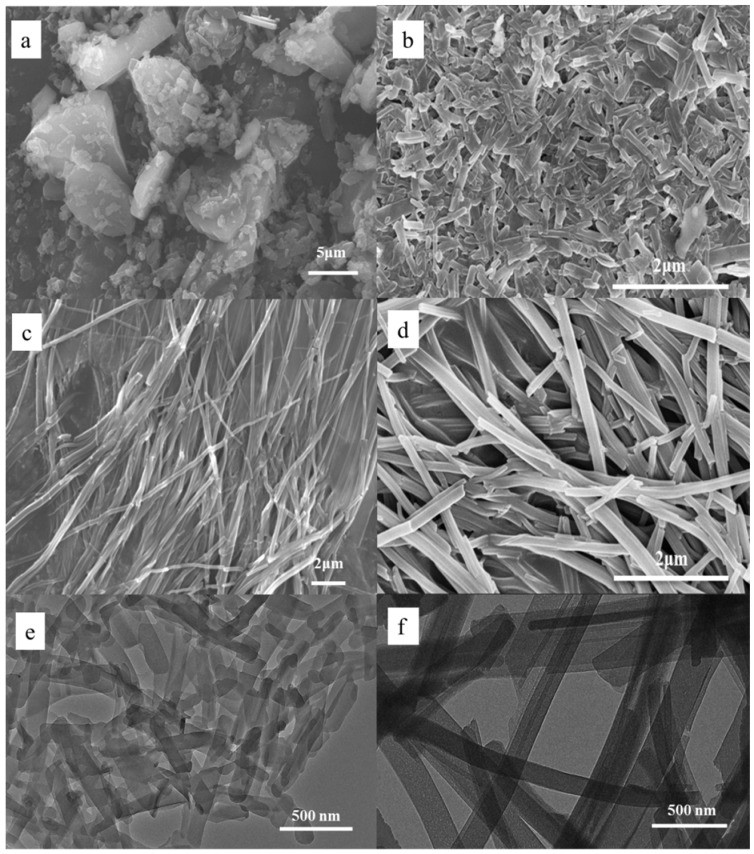
SEM of (**a**) untreated 5,10,15,20-tetrakis(4-carboxyphenyl)porphyrin (TCPP) powders; (**b**) hexadecyl trimethyl ammonium bromide (CTAB)-TCPP aggregates; (**c**) non-CTAB-TCPP aggregates (**d**) ethylene glycol (EG)-TCPP aggregates and TEM of (**e**) CTAB-TCPP aggregates (**f**) EG-TCPP aggregates.

Herein, surfactant-assisted self-assembly (SAS) was employed for the production of porphyrin supramolecular structures. Acid-based neutralization assisted with CTAB as a surfactant was used to synthesize assembled structures named CTAB-TCPP [21]. Generally, TCPP were deprotonated to form (TCPP)^−4^ in NaOH solution which was soluble in water. When (TCPP)^−4^ was injected into acidic aqueous solution in the presence of CTAB micelles, it was protonated and aggregated to form assembled structures due to the hydrophobic interactions of porphyrin rings. In another method, mixing dual-solvent systems containing good and poor solvents for TCPP under stabilizers EG was employed to form assembled structures named EG-TCPP [22]. The preparation procedure involved adding an excess volume of poor solvent (H_2_O) to a THF solution of TCPP in the presence of EG surfactant under stirring. Figure 1 shows the morphology of different assembly structures with different methods from SEM and TEM. Irregular bulks were observed through SEM for untreated TCPP powder. The morphology of CTAB-TCPP was estimated by TEM images and showed uniform nanorods with a width of ca. 125 nm and length of ca. 500 nm (Figure 1e). Dynamic light scattering (DLS) measurement was used to confirm the size of the aggregates (Figure S1) and it showed that the size of nanorods was in the range of 100 nm to 500 nm, which was in accordance with the TEM image (Figure 1e). Without CTAB surfactant, TCPP was precipitated using acid-based neutralization to form fibril structures with a length of more than 10 μm (Figure 1c). As shown in Figure 1d, the morphology of EG-TCPP aggregates showed nanofibers with diameters of ca. 250 nm and lengths of several micrometers. We observed from the SEM and TEM (Figure 1e,f) that the size of CTAB-TCPP aggregates was much smaller than that EG-TCPP. As we know, CTAB can form micelle structures with a hydrophobic cavity when its concentration is above critical micelle concentration. The hydrophobic cavity provided restricted space for protonated TCPP molecules to aggregate through intermolecular interactions. However, EG as stabilizing agents cannot provide the restricted space for TCPP aggregation, which resulted in different sizes and morphologies from CTAB-TCPP aggregates.

**Figure 2:**
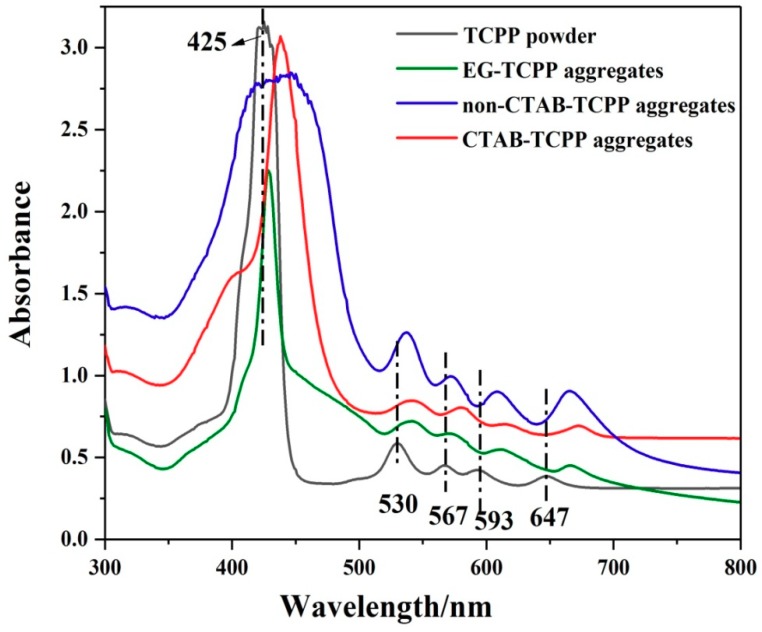
UV−vis absorption spectra of different TCPP aggregates and monomer.

To study the aggregation modes of TCPP molecules in aggregates, UV−vis absorption spectra of the different assemblies in the preparation medium were tested and are shown in Figure 2. TCPP molecules as monomer in N,N-Dimethylformamide (DMF) solution exhibited a typical Soret band at 425 nm and four Q bands at 530, 567, 593 and 647 nm [23]. For aggregated TCPP molecules of CTAB-TCPP, the Soret absorption band were red-shifted to 435 nm and the absorption peaks at Q bands also moved to long wavenumbers compared with TCPP monomer, which indicated that TCPP molecules aggregated in order through a J-type aggregation mode [24,25]. However, the absorption spectra of CTAB-TCPP aggregates showed a tiny shoulder band with high energy, implying the presence of an H-type aggregation mode between porphyrins. Without the CTAB, UV−vis absorption spectra of aggregated TCPP showed a broad Soret band and a strong shoulder band, which suggested that in the absence of CTAB micelles TCPP molecules assembled together disorderly with H-aggregation and J-aggregation coexisting in a similar proportion. For EG-TCPP aggregates, the Soret absorption band red-shifted slightly compared with TCPP monomer, which indicated that TCPP molecules in EG-TCPP assembly also aggregate via a J-type mode but with a lower degree of aggregation compared with that in CTAB-TCPP [26]. Herein, CTAB as a cationic surfactant was able to form micelles, which provided a restricted domain for aggregation of TCPP molecules via the non-covalent interactions, which facilitated porphyrins assembly mainly in the J-aggregation mode.

**Figure 3:**
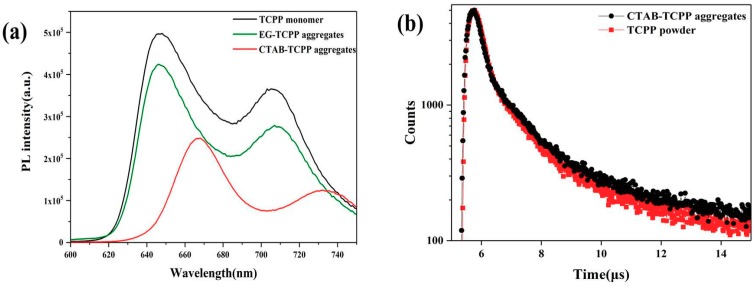
(**a**) The photoluminescence spectra and (**b**) time-resolved fluorescence decay curves of TCPP aggregates.

Figure 3a showed the photoluminescence spectra of the different aggregates. In the DMF solution, the TCPP molecules as a monomer showed emission bands of 648 and 703 nm, respectively [27], which is a characteristic pattern of porphyrin molecules. When forming CTAB-TCPP aggregates the two emission bands red-shifted and at the same time the bands were broadened. The red-shifting and widened emission band indicated the main J-aggregation mode of TCPP in CTAB-TCPP aggregates. However, there is no obvious red shifting in EG-TCPP aggregates, indicating the lower degree of aggregation in this assembly which leads to different photocatalytic activities. The time-resolved fluorescence decays of CTAB-TCPP aggregates and TCPP powders were investigated and the results are shown in Figure 3b. By using multi-exponential fitting, the average lifetimes of the TCPP aggregates were estimated and are shown in Table S1. The average decay time of CTAB-TCPP aggregates was shorter than that of TCPP powders, which confirmed the J-type aggregation mode of TCPP molecules [28] and more efficient separation of photogenerated carriers in CTAB-TCPP aggregates [29].

**Figure 4:**
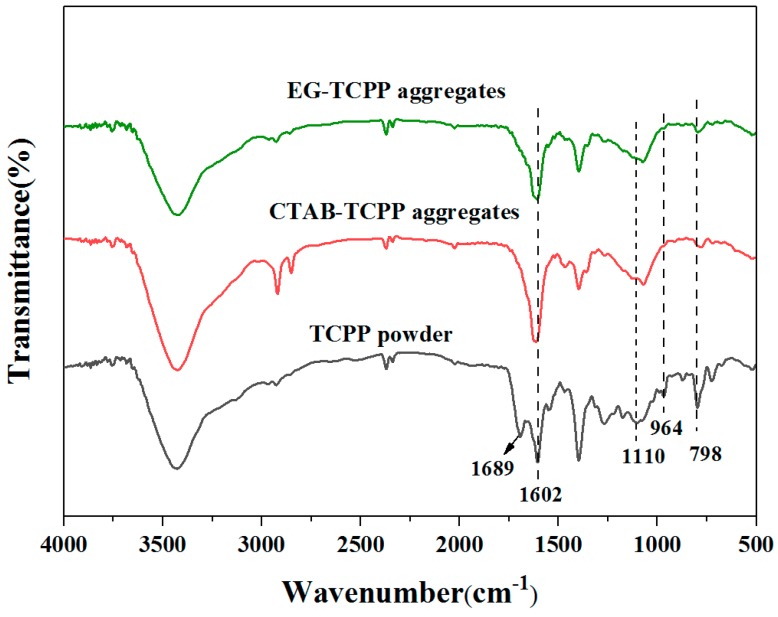
The FT-IR spectra of the different TCPP aggregates.

The FT-IR spectra of the different TCPP aggregates were measured to characterize the intermolecular interactions of TCPP molecules and shown in Figure 4. Compared with TCPP powders, the absorption band at 1689 cm^−1^ of C=O stretching vibration shifted to a lower wavenumber and overlapped with the vibration band at 1602 cm^−1^of aryl rings of TCPP. In addition, other absorption bands for stretching vibration of C–O and C–N at 1110 cm^−1^ and 798 cm^−1^ were all shifted to lower wavenumbers. All these results reveal the presence of hydrogen bond interactions between TCPP molecules in aggregates [30].

### 3.2. The Photocatalytic Activities of Different TCPP Aggregates

**Figure 5:**
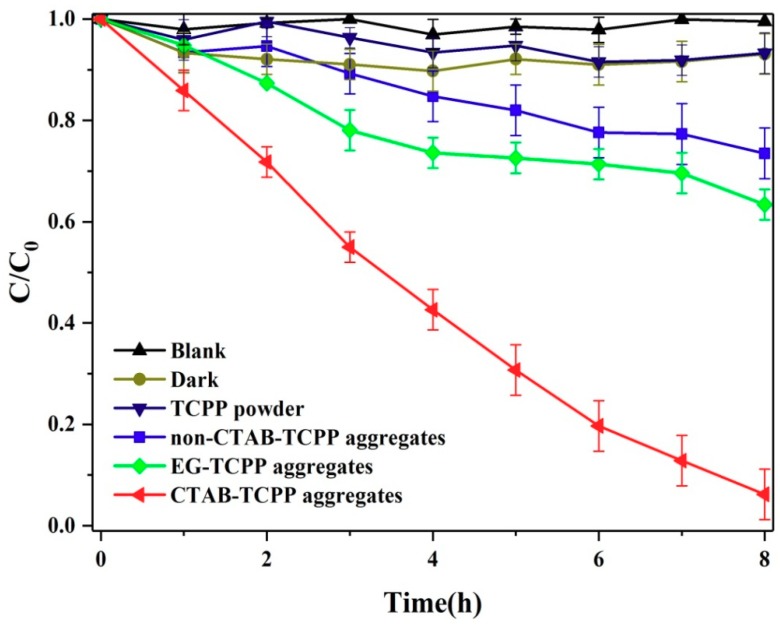
Degradation rates of phenol by different TCPP aggregates under visible light.

The photocatalytic activity of TCPP aggregates was tested for degradation of phenol under visible light irradiation. Figure 5 shows the degradation rates for phenol by different TCPP aggregates. No obvious photodegradation was observed by untreated TCPP powders under visible light irradiation. However, the calculated degradation rates of phenol were 94% and 36% by CTAB-TCPP aggregates and EG-TCPP aggregates, respectively. TCPP exists in the form of disordered molecules in untreated commercial powders and a very weak intermolecular interaction such as π−π stacking occurs, and hence, lower photocatalytic activity occurs compared to aggregated porphyrin molecules. In addition, compared to the EG-TCPP aggregates, CTAB-TCPP aggregates were superior photocatalysts for photodegradation of phenol, which proved that the photocatalytic activity of TCPP aggregates was related to the morphology of the aggregate structures as well as aggregation modes of TCPP molecules. It is noteworthy that just 10 mg of catalyst was used in this degradation process and compared with other organic photocatalysts such as the perylene-3,4,9,10-tetracarboxylic diimide (PDINH) supramolecular system and g-C_3_N_4_ as reported in the literature [5], its ability to degrade phenol was superior. In addition, our degradation experiment was performed under visible light and the degradation ability of other visible materials is incomparable [31].

The HPLC data for the phenol peak during the photodegradation by CTAB-TCPP aggregates is shown in Figure S2 in the Supplementary Materials and the retention time of phenol was 5.2 min. The peak of phenol reduced as the reaction proceeded and there were no other peaks. This indicates that phenols are mineralized to H_2_O and CO_2_ by CTAB-TCPP aggregates under irradiation. The three-dimensional chromatograms of phenol peaks at 0, 3, 6, and 8 h is given in Figure S3. The figure also shows that the characteristic peak of phenol gradually decreased as the time increased.

**Figure 6:**
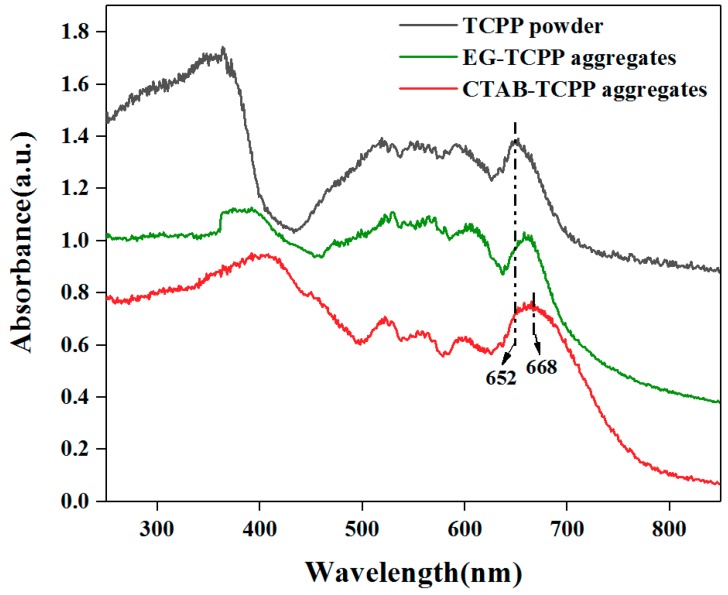
UV-Vis diffuse reflectance spectroscopy of different photocatalysts.

**Figure 7:**
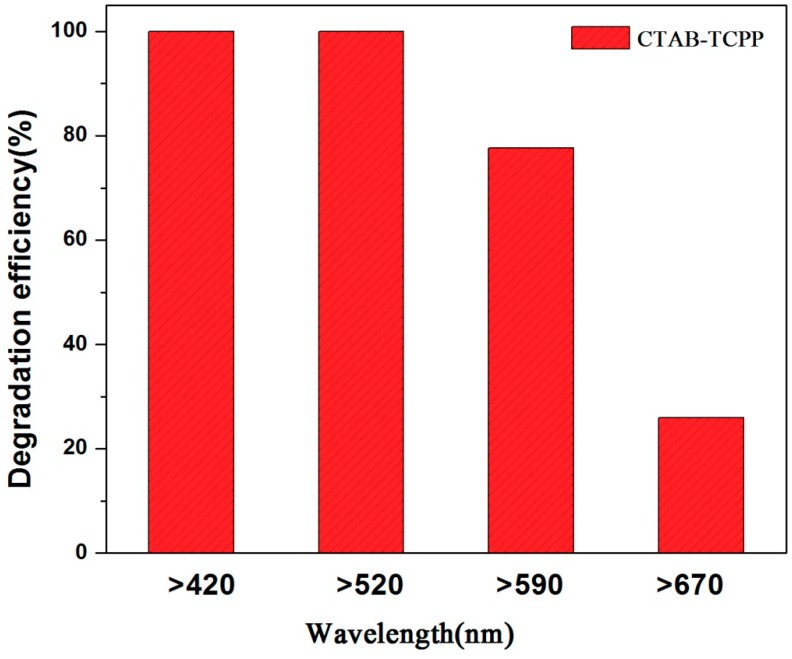
Degradation efficiencies by CTAB-TCPP under different wavelength ranges of light.

UV-vis diffuse reflectance spectroscopy (DRS) of different aggregates was measured to characterize the light absorption properties. Compared to untreated TCPP powders, the CTAB-TCPP aggregates exhibit a wider spectrum response, revealing their ability for wide range absorption of visible light (Figure 6). The absorption edge of CTAB-TCPP aggregates in the UV–vis spectrum expands to 668 nm and the photocatalytic reaction could be conducted under visible light irradiation. The properties of photodegradation of phenol under different light wavelengths were investigated and the results (Figure 7) showed that when the wavelength of irradiation light was above 520 nm, the photodegradation rate remained the same. When the wavelength of light was above 590 nm the degradation rate decreased to 85% and when it reached above 670 nm there was still degradation, which indicated that this self-assembled TCPP was efficient as a photocatalyst under a wide visible spectrum.

**Figure 8:**
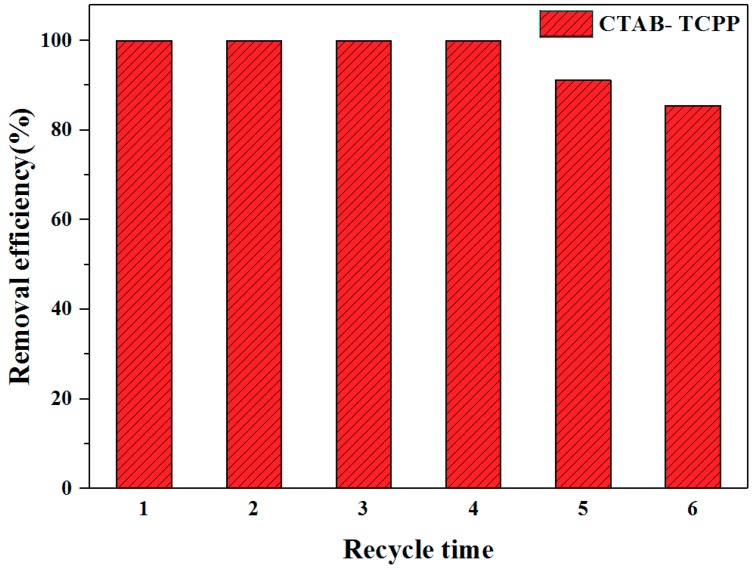
The cycling of degradation phenol by CTAB-TCPP.

The stability of photocatalysts is a critical factor for determining their applicability. Herein, the stability of CTAB-TCPP aggregates was investigated by observing several cycles of photodegradation of phenol. As shown in Figure 8, after four cycles the degradation efficiency of CTAB-TCPP aggregates started to be affected and was reduced to 87% after six cycles. The results indicated that the nonmetallic catalyst based on TCPP aggregates formed through non-covalent bond forces showed good stability and reusability during the degradation.

### 3.3. Mechanism of the Different Photocatalytic Activity of TCPP Aggregates

**Figure 9:**
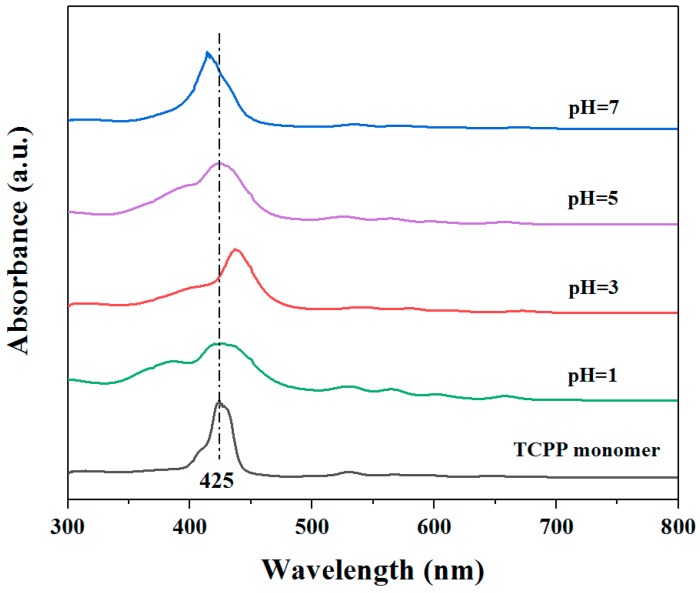
UV−vis absorption spectra of TCPP solution in N,N-Dimethylformamide (DMF) and CTAB-TCPP with different pH.

**Figure 10:**
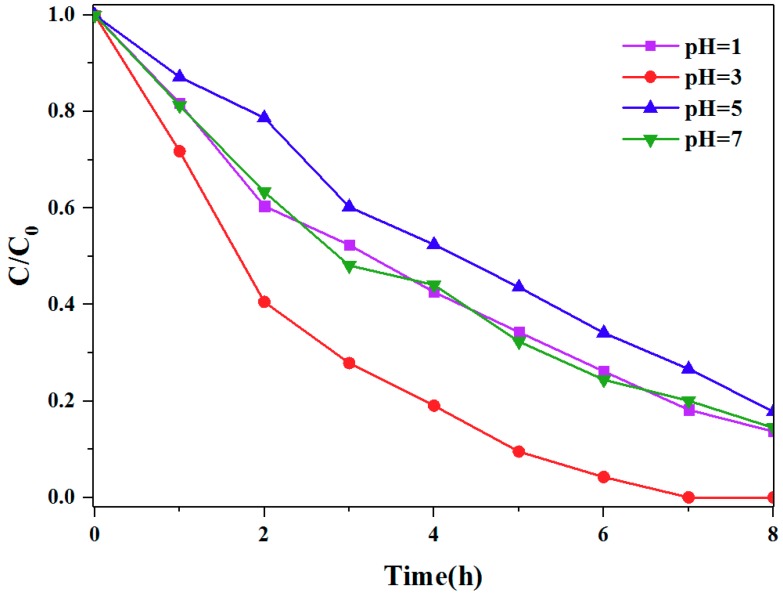
Activities of degradation phenol by CTAB-TCPP aggregates under different pH.

A possible mechanism has been suggested to explain the different photocatalytic activities performed by different TCPP aggregates. As observed above in the exploration of optical properties, in CTAB-TCPP assembly, TCPP molecules were aligned in a slipped cofacial mode as J-aggregation. In EG-TCPP assembly, TCPP molecules existed but with a lower degree of aggregation. As a result, CTAB-TCPP aggregates with rod shape TCPP molecules, showed greater π−π interactions between adjacent molecules than that of the EG-TCPP assembly. The J-aggregation mode of TCPP promoted delocalization of coherent π-electrons, which favors the electron transfer process and efficient photo semiconductor performance [32]. In contrast, the H-aggregated TCPP possessed a lower π conjugation structure and hence, it was not conducive to photocatalytic reactions. In order to prove this point, several CTAB-TCPP aggregates were prepared under different pH values regulated by HCl addition. In acid-based neutralization methods, different pH leads to different protonation degrees of the nitrogen atoms of the TCPP skeleton. The lower pH value resulted in protonation of pyrroles in the porphyrin core, which leads to greater repulsion between macro porphyrin rings; this is not conducive to face-to-face aggregation but to J-aggregation in CTAB micelles. At higher pH, nitrogen atoms are not protonated and the hydrophobic interaction between porphyrin rings is beneficial to face-to-face aggregation in CTAB micelles. This was proved by UV−vis absorption spectra of CTAB-TCPP aggregates with different pH and results are shown in Figure 9. At pH of 3, the Soret band of CTAB-TCPP aggregates was red-shifted compared with the TCPP monomer, indicating the J-aggregation mode of TCPP molecules. When pH was 7, the Soret band of CTAB-TCPP aggregates was blue-shifted, indicating TCPP molecules aggregated with the H-mode [33]. Correspondingly, the photocatalytic activities of CTAB-TCPP aggregates prepared with different pH values were different and as predicated, the CTAB-TCPP aggregates with a pH of 3 showed better degradation efficiency for phenol than those with a pH of 7 (Figure 10). If the solution was highly acidic with a pH of 1, strong repulsion between protonation TCPP molecules hindered their orderly alignment and the products presented the properties of a monomer. When the pH was 5, the UV−vis absorption spectra of aggregates were the same as a TCPP monomer, and it showed poor photocatalytic activity. The results demonstrated that the J-aggregation of TCPP through π−π interaction and hydrogen bonding interaction is beneficial to enhance the coherent electronic delocalization and charge separation, which enriches its photodegradation ability [34,35].

**Figure 11:**
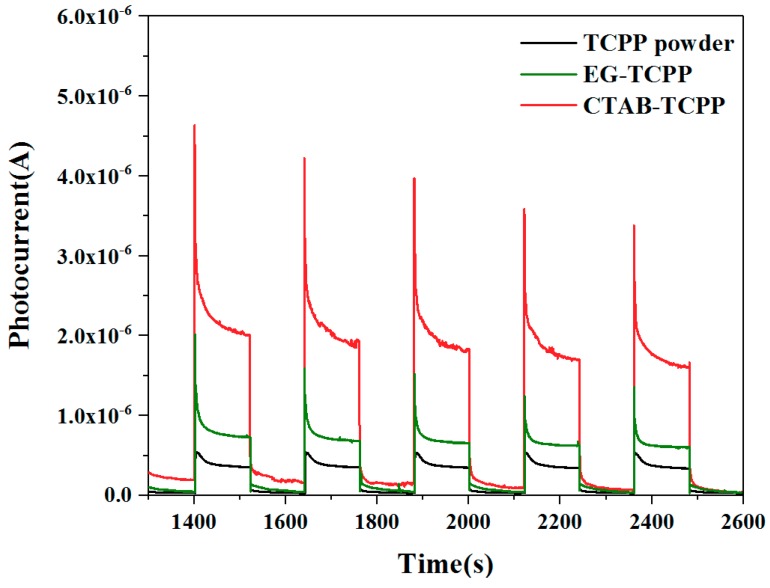
Transient photocurrent response spectra of the different TCPP aggregates.

**Figure 12:**
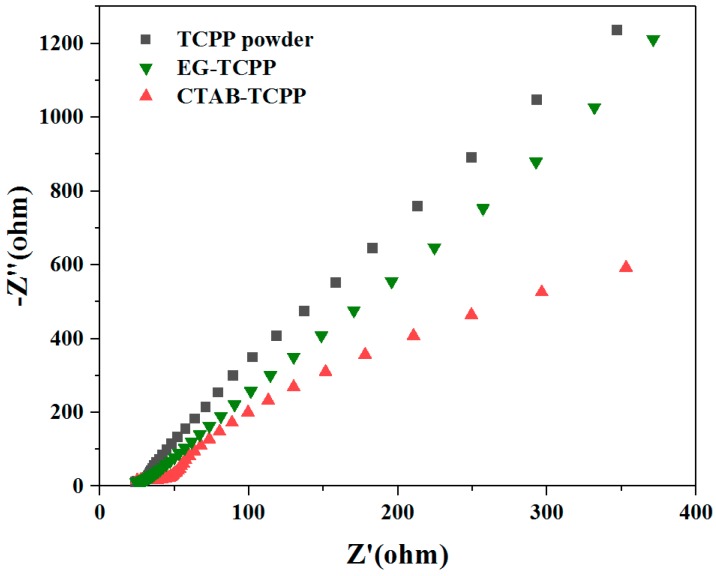
Electrochemical impedance spectra (EIS) of different TCPP aggregates.

Transient photocurrent response spectra of the photocatalysts can be exploited to study the electron-hole separation efficiency. As shown in Figure 11, the photocurrent of CTAB-TCPP aggregates and EG-TCPP aggregates were both higher than that of the untreated TCPP powders, which demonstrates the improved separation efficiency of the carriers in the assembly under visible light irradiation [36]. Moreover, the photocurrent of CTAB-TCPP aggregates was much stronger than that of EG-TCPP aggregates, which further reveals that the charge separation under light irradiation in CTAB-TCPP aggregates was more effective than that in EG-TCPP aggregates. Higher carrier separation efficiency leads to more photogenerated charges participating in the reaction, thus improving the degradation activity. Electrochemical impedance spectra (EIS) of different TCPP aggregates were tested to further demonstrate their electrical properties and are shown in Figure 12. The Nyquist curve radius of CTAB-TCPP aggregates and EG-TCPP aggregates were both lower than that of untreated TCPP powders, revealing that the resistance of photogenerated charge transfer was smaller in the TCPP assembly. At the same time, CTAB-TCPP aggregates have smaller charge transfer resistance and higher charge separation efficiency than EG-TCPP aggregates.

According to the absorption edge in the UV-vis diffuse reflectance spectra, the energy gaps of CTAB-TCPP aggregates and EG-TCPP aggregates were estimated to be 1.85 eV and 1.88 eV, respectively. For some organic aggregates, HOMO and LUMO of π-stacked molecules were overlapped and formed electronic energy level-like structures with conduction bands (CB) and valence bands (VB). Herein, the Mott-Schottky plots of CTAB-TCPP aggregates and EG-TCPP aggregates were used to determine the electronic energy levels and the results are shown in Figures S3 and S4 [37,38]. The flat band potential (Efb) for CTAB-TCPP aggregates and EG-TCPP aggregates were−1.04 eV and −0.76 eV (vs. SCE), respectively. So, the CB positions (HOMO level) of CTAB-TCPP aggregates and EG-TCPP aggregates was calculated to be −1.00 eV and −0.72 eV (vs. NHE). The CB position (HOMO level) of the EG-TCPP aggregates was lower than that of CTAB-TCPP aggregates. According to the energy gaps, the VB positions (LUMO levels) of CTAB-TCPP aggregates and EG-TCPP aggregates should be around +0.85 eV and +1.16 eV, respectively.

**Figure 13:**
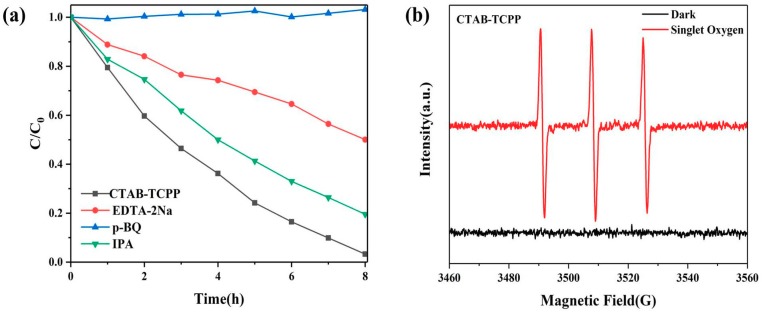
(**a**) Trapping experiments of reactive species by CTAB-TCPP aggregates (**b**) ESR spectra upon visible light irradiation of CTAB-TCPP aggregates for detection of ^1^O_2._

**Figure 14:**
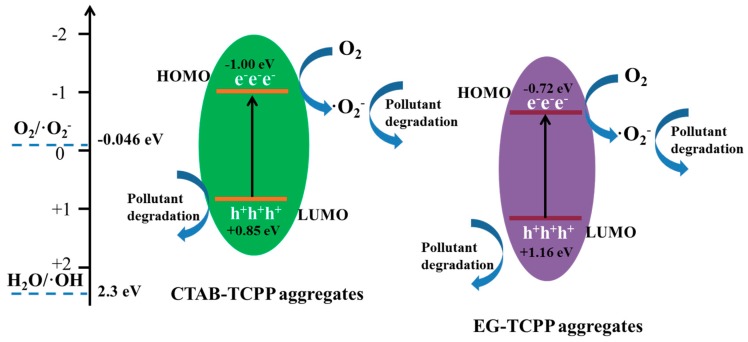
Predicted mechanism diagram of TCPP aggregates for photocatalytic degradation of phenol.

Trapping experiments were measured to test the active species of the photocatalysis reaction. Figure 13 shows the photodegradation rates for phenol by CTAB-TCPP aggregates in the presence of hole scavenger (EDTA-2Na) [39], superoxide radical scavenger (p-BQ) [40] and hydroxyl radical scavenger (IPA) [31], respectively. The degradation rate was reduced drastically in the presence of p-BQ, revealing that superoxide radical (•O_2_^−^) was the main oxidative species in the degradation reaction. In addition, the degradation rate was decreased in the presence of EDTA-2Na, proving that the holes (h^+^) also participated in the oxidation reaction. IPA had slight influence on the degradation of phenol, which suggested that a small fraction of hydroxyl radical (•OH) was produced in the degradation process. Meanwhile the electron paramagnetic resonance (ESR) with TEMP (2,2,6,6-tetramethyl-1-piperidine) as a spin probe was performed to detect the singlet oxygen (^1^O_2_) during irradiation of CTAB-TCPP aggregates. The ESR results in Figure 13b show that noticeable signals of ^1^O_2_ were observed, which revealed that ^1^O_2_ can be produced by CTAB-TCPP and also governs the photocatalytic degradation process. In view of the above discussion, a predicted photocatalytic mechanism of TCPP aggregates is shown in Figure 14. The photoexcited electrons reacted with O_2_ to generate •O_2_^−^, which oxidized phenol directly. In this process, hydrogen peroxide (H_2_O_2_) could be formed by O_2_with electrons and H^+^ and it further transformed into •OH [41] according to the theoretical potential value. The produced H_2_O_2_ and •OH were both detected with titanium (IV) oxysulfate and coumarin, respectively (Figure S5). Simultaneously, the h^+^ moves to the catalyst surface and oxidize the phenols. The different CB positions between EG-TCPP aggregates and CTAB-TCPP aggregates lead to different abilities to form superoxide radicals, which resulted in different photodegradation activities. Due to the more negative CB position (HOMO level) of CTAB-TCPP aggregates, the photogenerated electrons showed a greater ability to produce superoxide radicals. On the other hand, different aggregation modes of TCPP molecules and aggregate size resulted in the different separation efficiency of photogenerated carriers. Based on the factors discussed above, CTAB-TCPP aggregates present excellent photocatalytic activity for degradation of phenols.

## 4. Conclusions

In this paper, metal-free porphyrin (TCPP) was used to form supramolecular structures with orderly molecule aggregation and they showed enhanced photodegradation properties for phenol under visible light in contrast to untreated powders. In addition, the self-assembled aggregates of nanorods (CTAB-TCPP) with porphyrins in mainly J-aggregation mode demonstrated more efficient separation of photogenerated charges and a more positive CB position than that of nanofibers aggregates (EG-TCPP) through different assembly methods. Based on the above factors, CTAB-TCPP aggregates presented a strong ability to produce superoxide radicals as oxidative active species and showed excellent photocatalytic degradation activity. In conclusion, the metal-free porphyrin aggregates were proved to be a stable and effective photocatalyst for degradation of contaminants, while avoiding the introduction of metal ions. CTAB-TCPP aggregates have a high utilization rate of visible light, which is conducive to its practical application. This research may provide some theoretical guidance for the development of metal-free photocatalysts for use in the control of environmental pollution and solar energy conversion.

## Supplementary Materials

The following are available online at https://www.mdpi.com/2079-4991/9/9/1321/s1, Figure S1: Dynamic light scattering (DLS) of the CTAB-TCPP aggregates, Figure S2: High performance liquid chromatography of phenol peak, Figure S3: The three-dimensional chromatograms of phenol peak, Figure S4: The Mott-Schottky plots of CTAB-TCPP aggregates, Figure S5: The Mott-Schottky plots of EG-TCPP aggregates. Figure S6: The UV-vis adsorption and photoluminescence spectra of titanium (IV) oxysulfate and coumarin for detection of H_2_O_2_ and •OH. Table S1: Time resolved fluorescence fitting results of commercial TCPP and CTAB-TCPP.
