# Supplementary material for: Tuning the Supramolecular Structures of Metal-Free Porphyrin via Surfactant Assisted Self-Assembly to Enhance Photocatalytic Performance
Source: Nanomaterials (Basel). 2019 Sep 15;9(9):1321. doi: 10.3390/nano9091321 (PMC6781064; doi:10.3390/nano9091321)
Supplement: Supplementary file 1 [file nanomaterials-09-01321-s001.pdf]

Tuning the Supramolecular Structures of Metal-Free Porphyrin via Surfactant Assisted Self-Assembly to Enhance Photocatalytic Performance

Jinrong Lu, Zihan Li, Weijia An, Li Liu and Wenquan Cui *

College of Chemical Engineering, Hebei Key Laboratory for Environment Photocatalytic and Electrocatalytic Materials, North China University of Science and Technology, Tangshan 063210, China;

* Correspondence: wqcui@ncst.edu.cn

1. Dynamic light scattering (DLS) characterization

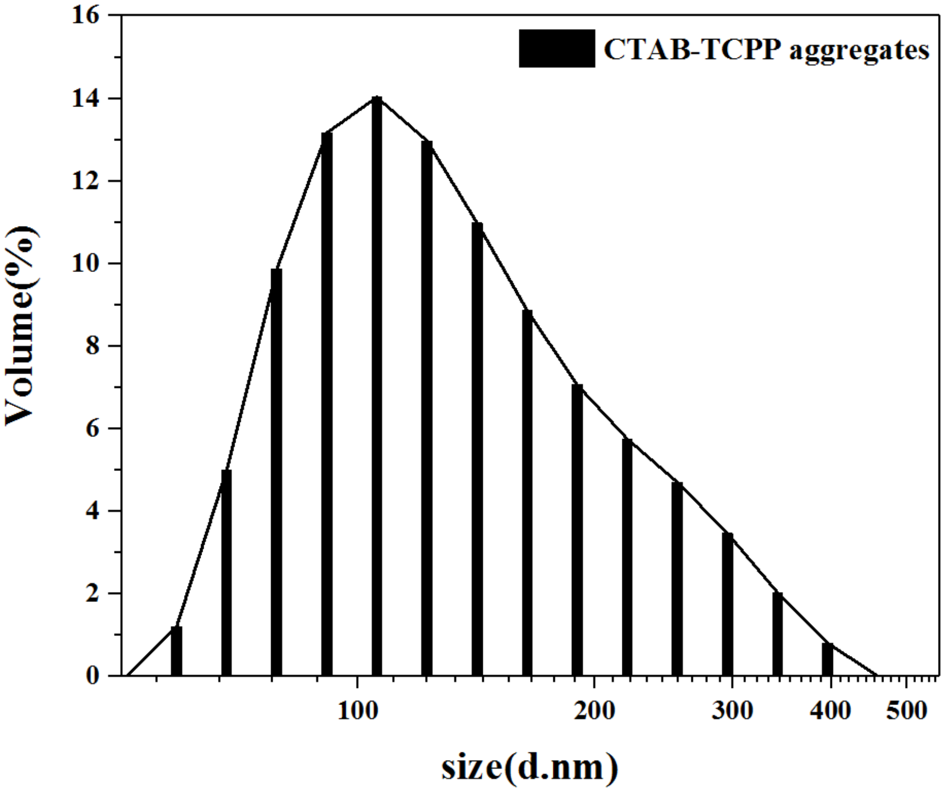

Figure S1. Dynamic light scattering (DLS) of the CTAB-TCPP aggregates.

2. The average of decay time of CTAB-TCPP aggregates

Table s1. Time resolved fluorescence fitting results of commercial TCPP、CTAB-TCPP

Sample	τ1(ns)	τ2(ns)	B1	B2	Average τ(ns)
Commercial TCPP	**773.14**	**2993.67**	**1133.602**	**465.384**	**2136.201**
CTAB-TCPP	**722.56**	**2610.284**	**1135.783**	**648.01**	**1993.604**

3. The HPLC data of phenol during the photodegradation

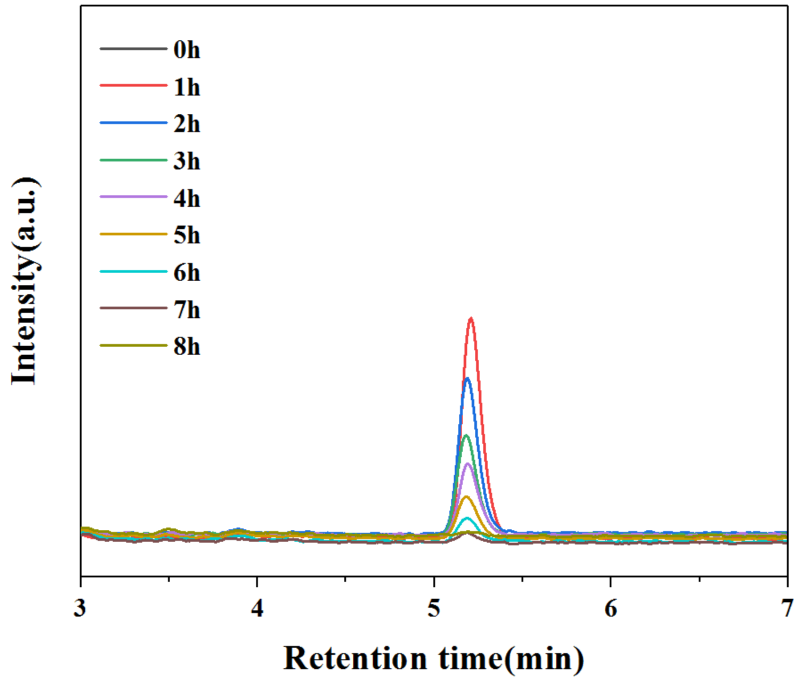

Figure S2. High performance liquid chromatography of phenol peak during degradation by CTAB-TCPP.

4. The three-dimensional chromatograms of phenol peak

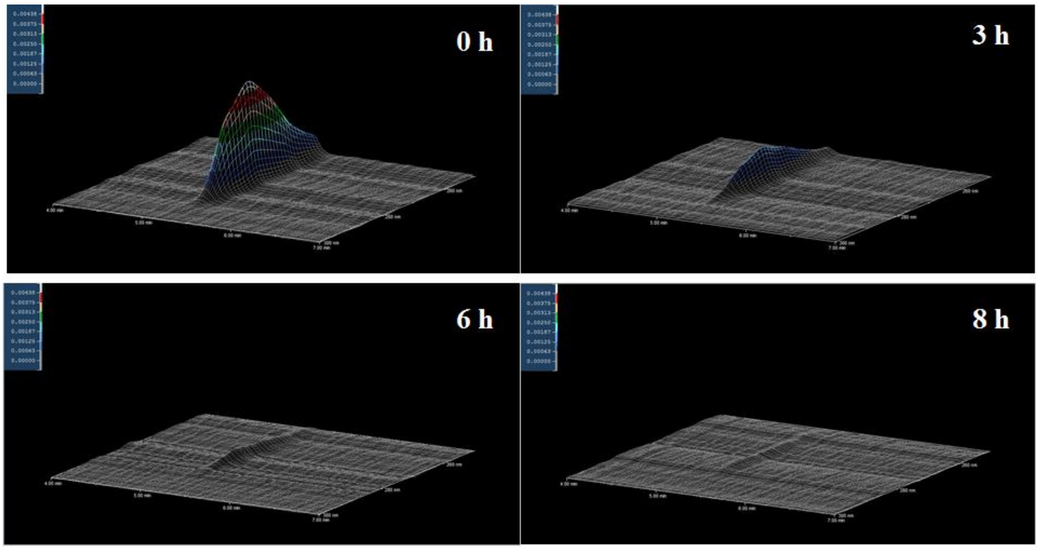

Figure S3. The three-dimensional chromatograms of phenol peak at 0, 3, 6 and 8 h.

5. The Mott-Schottky plots of CTAB-TCPP aggregates and EG-TCPP aggregates

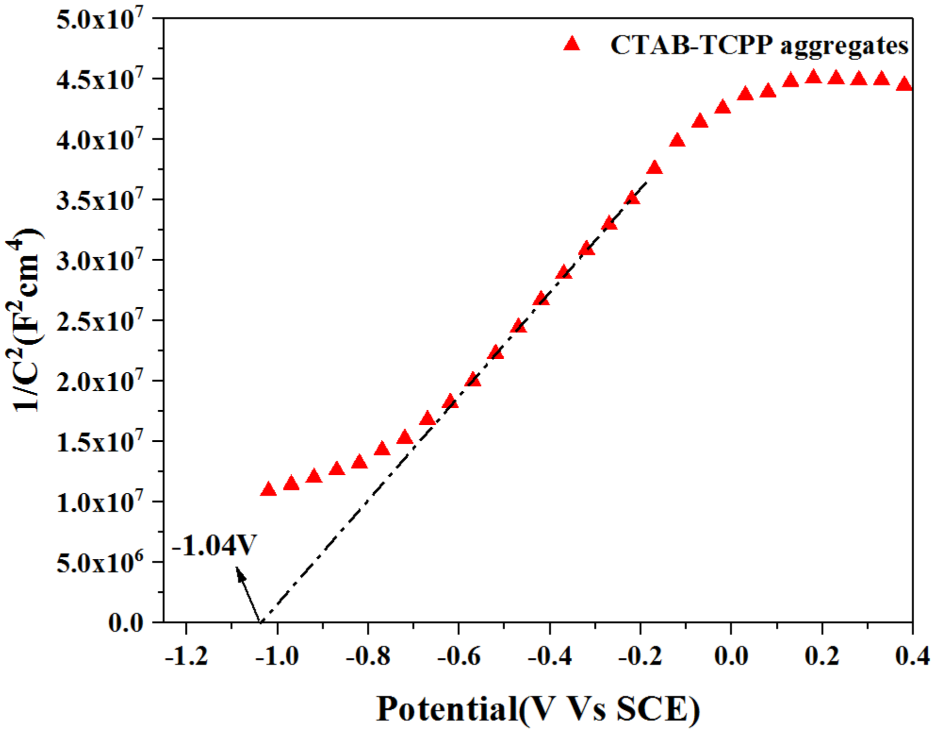

Figure S4. The Mott-Schottky plots of CTAB-TCPP aggregates. The frequnze of 1000 Hz was used to detect the flat band position.

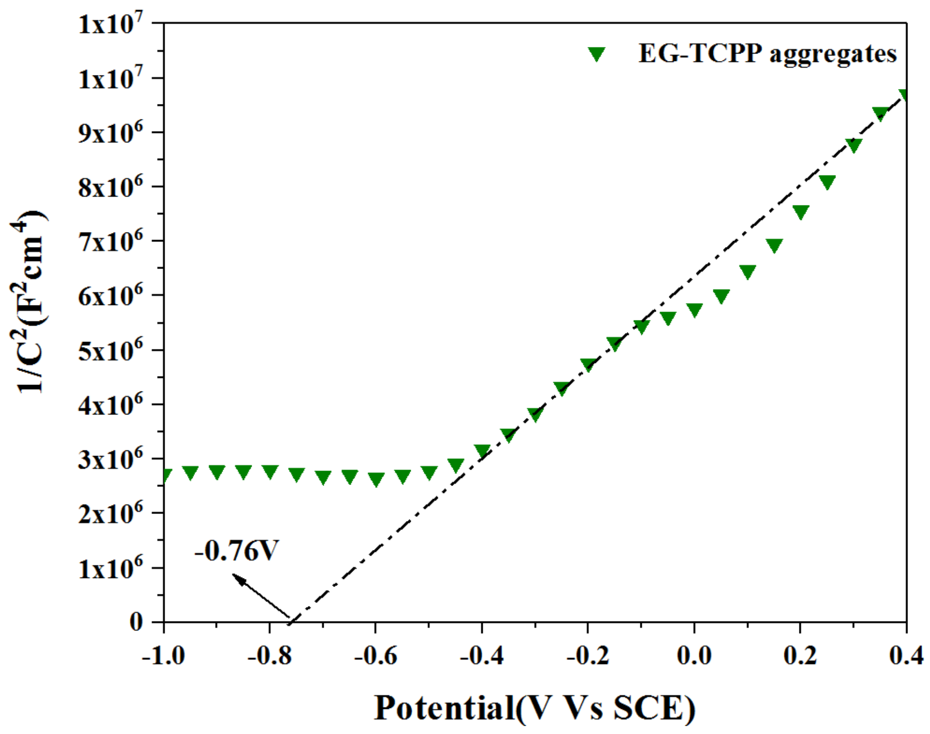

Figure S5. The Mott-Schottky plots of EG-TCPP aggregates. The frequency of 1000 Hz was used to detect the flat band position.

6. **The detection of produced H_2O_2 and •OH with titanium (IV) oxysulfate and coumarin characterized with UV-vis adsorption spectra and PL.**

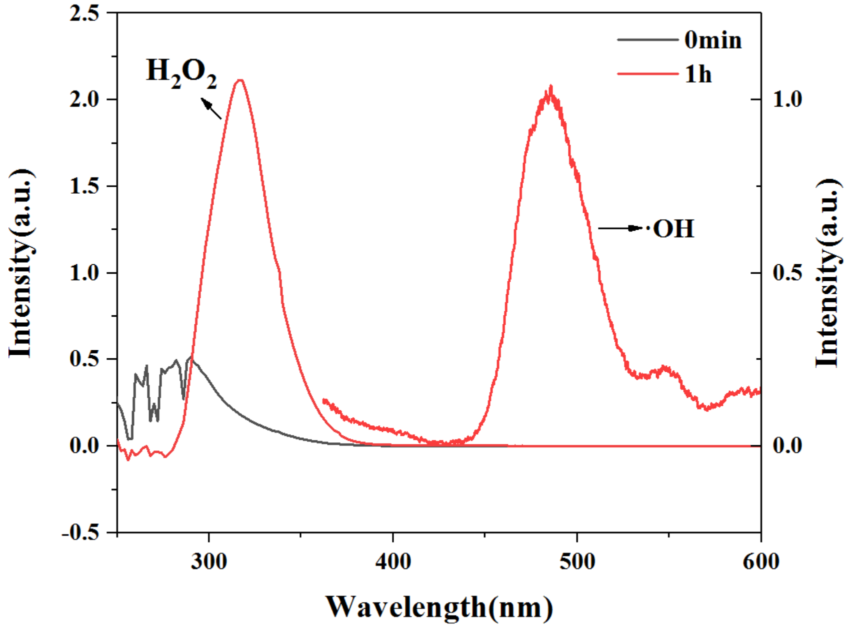

Figure S6. the UV-vis adsorption spectra and PL spectra of titanium (IV) oxysulfate and coumarin for detection of H_2O_2 and •OH.
